# Cardiac Adaptation in Power Athletes: Differential Impact of Judo and Weightlifting

**DOI:** 10.3390/jcm13113336

**Published:** 2024-06-05

**Authors:** Giuseppe Di Gioia, Armando Ferrera, Viviana Maestrini, Sara Monosilio, Maria Rosaria Squeo, Erika Lemme, Antonio Nenna, Sofia Calaciura Clarich, Simone Crotta, Antonio Pelliccia

**Affiliations:** 1 Institute of Sports Medicine and Science, National Italian Olympic Committee, Largo Piero Gabrielli, 1, 00197 Rome, Italy; armando.ferrera@uniroma1.it (A.F.); viviana.maestrini@uniroma1.it (V.M.); sara.monosilio@gmail.com (S.M.); mariarosaria.squeo@coni.it (M.R.S.); erikalemme@msn.com (E.L.); sofia.calaciuraclarich@uniroma1.it (S.C.C.); simone.crotta@uniroma1.it (S.C.); ant.pelliccia@gmail.com (A.P.); 2 Clinical and Molecular Medicine Department, Sapienza University of Rome, 00198 Rome, Italy; 3 Department of Heart Surgery, Fondazione Policlinico Universitario Campus Bio-Medico, Via Alvaro del Portillo, 200, 00128 Rome, Italy; 4 Department of Physiology and Pharmacology, Sapienza University of Rome, 00198 Rome, Italy

**Keywords:** power athletes, athlete’s heart, heart remodeling, elite, sport cardiology

## Abstract

**Background:** According to the ESC guidelines, sport disciplines are classified in relation to the predominant component (skill, power, mixed and endurance), including a wide range of disciplines with different isometric/isotonic exercises and exercise-induced heart remodeling. The aim of our study was to evaluate differences in morpho-functional cardiac adaptations in power athletes, comparing judokas with weightlifters. **Methods:** We enrolled 55 Olympic athletes (38 judokas, 17 weightlifters), aged 24.5 ± 3.8 years, 25 (45.4%) of whom were males, and they underwent a pre-participation evaluation, including a physical examination, ECG, transthoracic echocardiogram, and exercise stress test. **Results:** The judokas presented significant differences in cardiac adaptations, with larger left ventricle (LV) end-diastolic and end-systolic volumes indexed (LVEDVi, *p* = 0.002 and LVESVi, *p* = 0.004) and higher LVMass values indexed (*p* = 0.033), but similar LV wall thicknesses (*p* = 0.093) and LV ejection fractions (*p* = 0.981). Also, the left atrium (LA) dimension (*p* = 0.0002) and volume indexed (*p* < 0.0001) were higher in the judokas, as were the larger right ventricle (RV) areas. Finally, the judokas showed higher VO_2_max (*p* = 0.012), O_2_ pulse (*p* = 0.007), VE/O2 LT1 (*p* = 0.041) and VE/O2 LT2 (*p* = 0.036) values, with a lower resting heart rate (*p* = 0.031) and higher exercise capacity (*p* = 0.011). **Conclusions:** The judokas showed substantial differences in cardiac morpho-functional adaptations from the weightlifters, and, accordingly, judo should be more properly considered not a pure strength sport but more similar to mixed disciplines of the ESC classification.

## 1. Introduction

Cardiac adaptation to exercise training is a well-known phenomenon that typically includes increased left ventricular (LV) wall thickness and mass (hypertrophy), LV cavity enlargement (dilatation), and increased resting parasympathetic tone [1,2,3,4,5], which is commonly known as “athlete’s heart”, representing a physiological adaptation enabling athletes to increase their exercise performance [6]. Differences in cardiac remodeling have been described in relation to different types of exercise training [7]. Specifically, isotonic (dynamic) exercise, as performed by endurance athletes, is primarily characterized by increased stroke volume, reduced vascular resistance, and enlargement of the LV cavity dimensions with proportional wall thickening, termed eccentric hypertrophy (EC). Conversely, isometric (static) exercise, as performed by athletes practicing power disciplines with increments in blood pressure, is characterized by relative LV wall thickening without an increase in cavity dimensions [8] or evident cardiac remodeling [9].

Indeed, it should be considered that cardiac dimensions are also influenced by other factors, such as gender, body size, ethnicity, drug usage and genetics, other than the type of sporting discipline and extent of training [10]. Hence, LV geometry based on the ratio between the left ventricular mass index (LVMi) and the relative wall thickness (RWT) is used to better evaluate changes in LV remodeling in different types of sports [11].

The aim of our study was to evaluate differences in morpho-functional cardiac adaptations in power Olympic athletes, specifically comparing judokas with weightlifters. This comparison was triggered by the consideration that judokas exhibit great anaerobic capacity and upper body strength, but judo is also characterized by an aerobic component that helps them to have a quicker and more efficient recovery during short breaks between exertions [7,12].

Weightlifting is a quintessential power sport [7], requiring one to generate extremely high peak forces and contractile rates of force and, consequently, high peak power outputs and contractile impulses. Unlike judo, in weightlifting, which involves short pulses of exertion, the anaerobic component is totally dominant.

In light of the above, our hypothesis is that the differences in the endurance–strength balance between these two sports may lead to distinct patterns of cardiac remodeling, despite both being categorized as power disciplines.

## 2. Materials and Methods

The Institute of Sport Medicine and Science in Rome is a medical division overseen by the Italian National Olympic Committee, whose primary responsibility is to conduct medical evaluations of athletes selected to participate in events such as the Olympic Games, World Championships, and Mediterranean Games.

The research methodology employed in this investigation underwent scrutiny and approval by the Review Board of the Institute of Medicine and Sports Science. All athletes involved in this study were comprehensively apprised of the nature and scope of the evaluation and provided their informed consent in compliance with Italian law and the institute’s protocols. The entirety of the clinical data compiled from the study cohort are cataloged within an institutional database. The activities detailed herein were conducted in accordance with the Code of Ethics of the World Medical Association (Declaration of Helsinki).

We enrolled 55 Olympic power athletes who participated in the London 2012, Rio 2016 and Tokyo 2020 Summer Olympic Games. The athletes underwent a comprehensive, multidisciplinary pre-participation screening, which included a complete physical examination, resting electrocardiography (ECG), transthoracic echocardiography (TTE), and a maximal exercise stress test.

Blood pressure was measured in the sitting position before exercise testing, as recommended [13].

Body height and weight were obtained for each subject, and body mass index (BMI) was calculated as weight (kg)/height (m^2^). Body surface area (BSA) was derived from the Mosteller formula [14]. Body composition and fat mass percentage were measured using Bioelectric Impedance Analysis (BIA 101 Quantum, Akern, Italy) using a constant sinusoidal current at an intensity of 50 kHz and 400 μA. Standard 12-lead ECG was performed in supine position, and an interpretation was made according to the international criteria for ECG interpretation in athletes [15]. All participants underwent maximal exercise testing on a bicycle ergometer (Cubestress XR400; Cardioline SpA, Milan, Italy) as previously reported with an incremental protocol until exhaustion.

## 3. Cardiopulmonary Exercise Test (CPET)

Maximal oxygen uptake (VO_2_max) was determined by a ramp incremental exercise test [16] with a starting load of 30 W and a continuous incremental load of 1 W every 3 s (20 W/min), maintaining a speed of 60 rpm till exhaustion on a bicycle ergometer. When the participant could no longer complete the desired workload, the test was terminated. Oxygen uptake was measured continuously with an automated system (Cosmed, Albano Laziale, Italy). The exercise test was accepted as maximal if the majority of the following termination criteria were met: respiratory exchange ratio greater than 1.0, heart rate greater than 99% of the maximum predicted value, or indicators of a maximal effort such as sweating or when, despite strong verbal encouragement, the participant was unable or unwilling to continue.

## 4. Transthoracic Echocardiogram

The echocardiographic assessment was performed on subjects in a resting state, positioned in the left lateral decubitus orientation.

Ultrasound data acquisition was performed utilizing a GE Vivid E9 ultrasound system equipped with a 4Vc phased array probe (GE Healthcare Vingmed Ultrasound AS, Horten, Norway). A comprehensive 2D echocardiographic study was carried out, wherein cardiac images were captured in various cross-sectional planes employing established transducer positions. Conforming to the current recommendations [17], measurements of the LV end-diastolic diameter (LVEDD), the left ventricle end-systolic diameter (LVESD), the IVS thickness and the PWT were taken in the parasternal short-axis section of the LV. The RWT was calculated using the ratio of (IVS + PWT) to LVEDD [17]. LV mass (LVM) was derived using the Devereux formula [11], with measurements indexed to body surface area (BSA) [18]. An LVMi > 115 g/m^2^ for males and >95 g/m^2^ for females was indicative of LV hypertrophy [18]. Different types of LV remodeling [17] were defined based on the measurements obtained, including normal geometry (NG), defined as an LVM ≤ 115 g/m^2^ in males or ≤95 g/m^2^ in females and an RWT ≤ 0.42; concentric remodeling (CR), defined as an LVM ≤ 115 g/m^2^ in males or ≤95 g/m^2^ in females and an RWT > 0.42; concentric hypertrophy (CH), defined as an LVM > 115 g/m^2^ in males or >95 g/m^2^ in females and an RWT > 0.42; and eccentric remodeling (ER), defined as an LVM > 115 g/m^2^ in males or >95 g/m^2^ in females and an RWT ≤ 0.42.

LV systolic function was assessed based on the LV ejection fraction (EF), derived from the LV end-diastolic volume (LVEDV) and end-systolic volume (LVESV), computed through LV biplane planimetry employing the modified Simpson’s rule in both the apical two- and four-chamber views [18]. Diastolic function was evaluated using both pulsed-wave Doppler (PW) and tissue Doppler imaging (TDI) as recommended [19], based on the measurement of the maximum E- and A-wave blood flow velocities (Vmax), the E/A-ratio, and the myocardial Vmax of e′ and a′ at the basal septal and lateral tricuspid annulus, along with the septal E/e′-ratio [19]. The left atrial (LA) volume was determined using the biplane method. The right ventricular (RV) chamber was evaluated in accordance with established guidelines [20]. The right atrial (RA) area and RV function parameters were assessed in the RV-focused apical four-chamber view by tracing the endocardial contour of the end-diastolic and systolic areas, from which the fractional area change (FAC) was calculated and expressed as a percentage [20]. Tricuspid annular plane systolic excursion (TAPSE) was measured as an indicator of RV longitudinal systolic function [20]. The peak tricuspid regurgitant velocity and the systolic trans-tricuspid gradient were determined using continuous-wave Doppler imaging on the tricuspid regurgitation jet. The pulmonary artery systolic pressure (PASP) was calculated by adding the systolic trans-tricuspid gradient to the value of the right atrial pressure (RAP). The latter was estimated using the inferior vena cava (IVC) dimension, inspiratory collapsibility, and RV function [20]. In all athletes, three consecutive cardiac cycles were assessed in accordance with the current literature [21].

All echocardiographic measurements were executed by experienced sports cardiologists (GDG and AP). From 2012 to 2016, echocardiograms were exclusively performed by a single expert sport cardiologist (AP). From 2016 to 2022, a second physician (GDG) joined to the team and started performing echocardiograms. However, before this, to validate reports, all echocardiograms were re-evaluated by AP. Intra-observer and inter-observer variability were therefore assessed. The two investigators (GDG and AP) blind-measured the same exam. Both the investigators repeated the analysis 2 days later, blinded to the previous measurements. The interclass correlation coefficients (ICCs) for the LVEF were 0.91 for intra-observer agreement and 0.92 for inter-observer agreement; for the LVEDD, the ICC was 0.93 for intra-observer agreement and 0.92 for inter-observer agreement; for the IVS thickness, the ICC was 0.94 for intra-observer agreement and 0.93 for inter-observer agreement; for the LVEDV, the ICC was 0.90 for intra-observer agreement and 0.89 for inter-observer agreement. Discrepancies between observers were resolved by consensus.

## 5. Statistical Analysis

Categorical variables were expressed as frequencies and percentages and were compared using Fisher’s exact test or the Chi-square test, as appropriate. Normality criteria were checked for any continuous variable, which was presented as the mean and standard deviation (SD) and compared using the Student’s *t*-test for independent data if normally distributed. Pearson’s correlation coefficient was used for the correlation analysis. All tests were considered significant if *p* < 0.05. ICCs were calculated to assess the inter-observer and intra-observer agreement for the main left ventricle measurements.

The statistical analysis was performed with STATA Statistics for Windows (SE, version 17) software.

## 6. Results

Of the 55 Olympic athletes included in this study, 38 (69.1%) practiced judo and 17 (30.9%) practiced weightlifting; the mean age was 24.5 ± 3.8 years, and 25 (45.4%) were males, with a mean BMI of 25.6 ± 3.8. All athletes were Caucasians. Seven (12.7%) were smokers, and nine (16.4%) had familiarity with cardiovascular disease. Two female athletes were taking estrogen hormones, one athlete was taking iron supplements, and one athlete was using a bronchodilator for asthma.

The judokas presented significantly more hours per week of training (22.3 ± 3.6) compared to the weightlifters (13.2 ± 2.8, *p* < 0.0001).

Differences in anthropometric parameters, echocardiographic measurements and exercise stress tests between the athletes practicing judo and the weightlifters are highlighted in Table 1.

No significant differences were found in terms of male prevalence (*p* = 0.875), age (*p* = 0.243), weight (*p* = 0.101), BMI (0.622) and fat mass % (*p* = 0.307), with the judoka athletes presenting a higher BSA, close to statistical significance (*p* = 0.058). The global amount of training hours/week was also similar (25.3 ± 8.9 in judokas vs. 21.7 ± 8 in weightlifters, *p* = 0.267).

The athletes practicing judo presented significant differences in cardiac remodeling, with a higher LVEDVi (66 ± 17.4 mL/m^2^ vs. 48.2 ± 20.8 mL/m^2^, *p* = 0.002) and LVESVi (23.3 ± 7.4 mL/m^2^ vs. 16.7 ± 7.3 mL/m^2^, *p* = 0.004) and, consequently, a higher LVMi (94.3 ± 19.6 g/m^2^ vs. 82.8 ± 12.4 g/m^2^, *p* = 0.033), but they showed similar LV wall thicknesses (IVS, *p* = 0.087 and PWT, *p* = 0.093) and systolic function as expressed by LV EF (*p* = 0.981). A larger atrial remodeling was also noted, with a larger diameter (36 ± 4.6 mm vs. 30.7 ± 4.1 mm, *p* = 0.0002) and volume index (LAVi: 23.3 ± 5.7 mL/m^2^ vs. 15.7 ± 3.3 mL/m^2^, *p* < 0.0001). A greater RV area was also observed in the athletes practicing judo (RVEDA 24.3 ± 4.2 mm^2^ vs. 19.7 ± 4.3 mm^2^, *p* = 0.005 and RVESA 12.9 ± 2.9 mm^2^ vs. 10.7 ± 2.4 mm^2^, *p* = 0.033), with a similar FAC (*p* = 0.583). Finally, no differences were noted in the indexes of diastolic function and the TDI velocities.

Finally, the type of LV remodeling showed differences (Table 2). All the athletes practicing weightlifting presented an NG and none had an EH, CR, or CH. The majority of those practicing judo (73.7%, *p* = 0.019) had an NG, while most of the remaining had ER (n = 9, 23.7%, *p* = 0.028). No gender differences in ER remodeling were noted (male, 29.4% vs. female, 19%; *p* = 0.454).

In the exercise stress test, the athletes practicing judo had a lower resting heart rate (67.2 ± 13.8 bpm vs. 76.8 ± 12.7 bpm, *p* = 0.031) and mildly higher blood pressure values (rest SBP, *p* = 0.053; rest DBP, *p* = 0.035; and peak SBP, *p* = 0.0002) but a higher exercise capacity (2.98 ± 0.5 watt/kg vs. 2.53 ± 0.7 watt/kg, *p* = 0.011).

Moreover, in 31 athletes (24 judo and 7 weightlifting), a CPET was performed (Table 3). Overall, the judokas showed a higher VO_2_max (41.8 ± 5.2 mL/kg vs. 35.1 ± 5.4 mL/kg, *p* = 0.012), a higher O_2_ pulse (19.2 ± 3.3 vs. 14.8 ± 2.7, *p* = 0.007), and higher VE/O2 LT1 (1955.7 ± 443 mL/min vs. 1828.7 ± 770.5 mL/min, *p* = 0.041) and VE/O2 LT2 (2750.3 ± 511.9 mL/min vs. 2112 ± 399.7 mL/min, *p* = 0.036) ratios, with similar VE/CO2 values at LT1 and LT2 (respectively, *p* = 0.070 and *p* = 0.129).

## 7. Discussion

The concept of cardiac remodeling in athletes was pioneered by John Morganroth in 1975, when he introduced the concept of type-specific cardiac remodeling. Athletes participating primarily in isotonic disciplines were described as presenting an increase in LVEDV with little or no increase in LV wall thickness, whereas those practicing primarily isometric exercise had an increase in LV wall thickness associated with normal LVEDV [22].

In the following years, doubt was cast upon this concept. In 1994, Spirito et al. [23] reported the morphology of the “athlete’s heart” assessed by echocardiography in 947 elite athletes representing 27 different sports, highlighting that the development of cardiac remodeling was not to be considered a dichotomous concept but that sports associated with greater LV cavity dilation also presented a large extent of wall thickening [24,25].

In 2000, a meta-analysis by Pluim et al. [26] made a remark on this concept, showing that although divergent cardiac adaptations in dynamic and static sports may exist, there is an intermediate pattern in sports with high static and dynamic components. Ultimately, the 2020 European Society of Cardiology Guidelines [7] classified sports in relation to the predominant component (skill, power, mixed and endurance) and intensity of exercise (low, medium and high). In this context, it is obvious that a precise classification of all sports is extremely difficult because of the differences in the type of muscular work, the mode, volume and intensity of exercise, and an individual athlete’s response to a training stimulus. Moreover, in most sports, both isotonic and isometric muscular components co-exist. Certain sports demand a significant motor control element and a high level of skill, while others are characterized by varying intensities of exercise, ranging from low to very high. These intensity levels may be contingent upon the nature of the sport or the athlete’s level, whether professional, amateur or recreational.

In this setting, judo and weightlifting are considered two power sports, even if the first one is characterized by dynamic, high-intensity and intermittent exercise, similarly to that seen in certain aerobic disciplines. On the other hand, weightlifters’ training generates extremely high peak forces and contractile rates of force development and, consequently, high peak power outputs and contractile impulses.

In our study, we observed that highly trained judokas present notable differences in heart morphology when compared to weightlifters. Specifically, the internal dimensions of the heart, including the LVEDVi, LVESVi, LAVi, RV EDA, and RV ESA, were significantly larger in the judokas compared to the weightlifters. The judokas also exhibited increased LVM. Nine of them showed LV ER, while the others maintained an NG, likely as a result of a combination of aerobic and anaerobic exercise training. However, no differences in systolic and diastolic heart function between the judokas and weightlifters were found, and all these parameters were within normal ranges.

**Figure 1:**
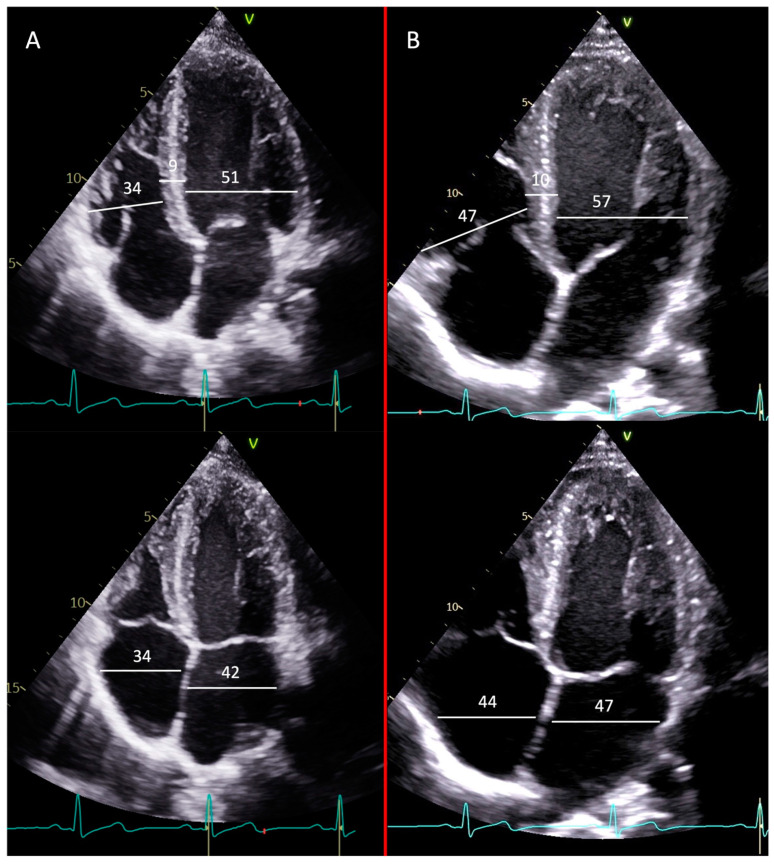
Transthoracic echocardiogram, apical four chamber view; upper figures: diastole; lower figures: systole. Panel (**A**): weightlifter. Panel (**B**): judoka. The athlete practicing judo presented larger diameters for both the ventricle and atria.

Judo, being a sport involving isometric exercises, has previously been reported to be associated with LV wall thickness values of up to 13 mm [27]. In our study, however, all the judoka athletes had wall thicknesses of <11 mm. Also, the RWT did not significantly differ between the two groups. Figure 1 shows the echocardiographic differences between two Olympic athletes practicing weightlifting and judo, respectively.

Moreover, in the weightlifter group, no athletes showed CH, and all of them (n = 17) had an NG, suggesting that weightlifters’ training may not always be sufficient to induce LV hypertrophy and any consequent cardiac remodeling.

**Figure 2:**
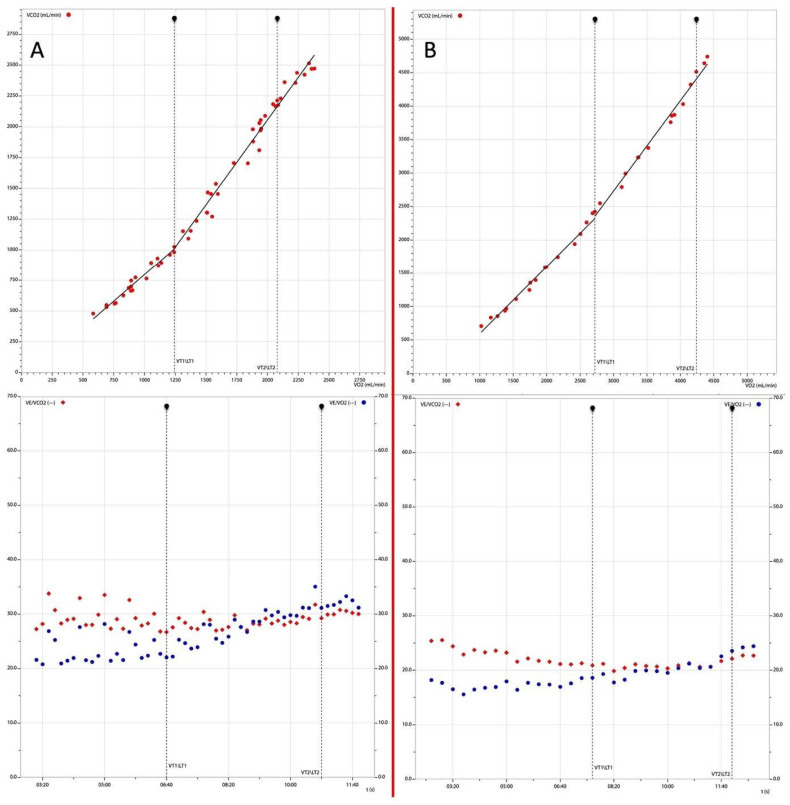
CPET comparison between athletes practicing weightlifting (Panel (**A**)) and judoka (Panel (**B**)). Judoka presented a higher VE/VO2 at both thresholds (LT1 and LT2) and a lower VE/CO2 ratio.

The existence of ER in a large subset of the judokas was also supported by the results of the CPET, which showed higher values of VO2, O2 pulse and VE/VO2 at both thresholds (LT1 and LT2) in the CPET than weightlifters (Figure 2).

Therefore, our study highlights that judokas exhibit a type of cardiac remodeling that is intermediate between the endurance athletes and purely power athletes, suggesting that this discipline should be more likely included into mixed disciplines in the ESC classification.

## 8. Limitations

Our study has some limitations that warrant acknowledgment: Firstly, its retrospective design. Secondly, although all the athletes were at an Olympic level, the sample size was notably small, potentially limiting the statistical power and generalizability of the findings. Then, the study population comprised exclusively Caucasians, thus potentially restricting the applicability of the results to other ethnic groups due to variations in physiological responses across different ethnicities. These limitations underscore the need for caution in interpreting the study’s conclusions and highlight the importance of future research endeavors incorporating larger, more diverse cohorts and employing prospective study designs to corroborate and expand upon the current findings.

Finally, despite the fact that this study was focused on Italian Olympic athletes, who are subject to rigorous and continuous anti-doping testing, powerlifting has been associated in previous studies with a higher prevalence of doping cases [28,29,30].

While this study does not directly address the issue of performance-enhancing substance use, it is important to acknowledge that such practices could potentially confound the interpretation of cardiac remodeling patterns in athletes.

However, it is crucial to emphasize that the findings and heart remodeling may not be directly influenced by doping practices. Nonetheless, the potential impact of performance-enhancing substance use on an athlete’s heart remains an important consideration and warrants further investigation in future studies.

## 9. Conclusions

The 2020 ESC classification guidelines on Sports Cardiology include in power sports a wide range of disciplines with different isometric/isotonic ratios and different exercise-induced cardiac remodeling. In our study, comparing judo and weightlifting, we observed that judo is associated with a mildly enlarged LV cavity and increased LV mass, LA size and RV cavity, suggesting cardiac remodeling that is different from that seen in purely strength-trained athletes, such as weightlifters.

According to our study’s results, judo should be more properly considered as not a pure strength type of sport but more similar to the mixed sport disciplines of the ESC 2020 classification.

## Figures and Tables

**Table 1:** Anthropometric parameters, echocardiographic measurements and exercise stress test differences between athletes practicing judo and weightlifters.

N = 55	Weightlifting	Judo	*p*
n (%)	17 (30.9)	38 (69.1)	-
Male, (%)	8 (47.1)	17 (44.7)	0.875
Age, years	23.6 ± 4.6	24.9 ± 3.3	0.243
Weight, kg	67.2 ± 13	75.5 ± 18.2	0.101
BMI, kg/m^2^	25.2 ± 3.1	25.7 ± 4.1	0.622
BSA	1.73 ± 0.2	1.87 ± 0.3	0.058
Fat mass, %	14.8 ± 4.6	17 ± 7.4	0.307
Training, hours	21.7 ± 8	25.3 ± 8.9	0.267
LV EDDi, mm/m^2^	28 ± 2	27.8 ± 2.2	0.702
LV ESDi, mm/m^2^	17.8 ± 1.5	17.3 ± 3.3	0.537
LV EDVi, mL/m^2^	48.2 ± 20.8	66 ± 17.4	0.002
LV ESVi, mL/m^2^	16.7 ± 7.3	23.3 ± 7.4	0.004
LVMi, gr/m^2^	82.8 ± 12.4	94.3 ± 19.6	0.033
IVS, mm	8.82 ± 1.0	9.4 ± 1.3	0.087
PWT, mm	8.58 ± 0.9	9.2 ± 1.3	0.093
RWT	0.35 ± 0.02	0.35 ± 0.03	0.923
LAVi, mL/m^2^	15.7 ± 3.3	23.3 ± 5.7	<0.0001
EF, %	64.6 ± 4.9	64.7 ± 5.6	0.981
LA, mm	30.7 ± 4.1	36 ± 4.6	0.0002
AR, mm	27.3 ± 3.7	29.6 ± 3.6	0.041
AA, mm	25.8 ± 4	25.8 ± 3.1	0.980
E wave, cm/s	88.8 ± 9.5	88.6 ± 15.2	0.974
A wave, cm/s	50 ± 10.6	47.8 ± 12.9	0.552
E/A	1.87 ± 0.5	1.94 ± 0.5	0.614
E’/A’	2.28 ± 0.7	2.06 ± 0.5	0.209
E/E’	7.4 ± 1	7.3 ± 1.4	0.692
E’, m/s	12.2 ± 1.8	12.5 ± 1.8	0.593
A’, m/s	5.7 ± 1.3	6.3 ± 1.4	0.148
S’, m/s	7.4 ± 0.9	7.2 ± 1.3	0.768
RVOTi LAX, mm/m^2^	16.7 ± 2.9	15.4 ± 2.2	0.091
PASP, mmHg	21.9 ± 3.2	22.8 ± 3.1	0.098
TAPSE, mm	27.1 ± 5.9	27.7 ± 4.2	0.334
RV EDA, mm^2^	19.7 ± 4.3	24.3 ± 4.2	0.005
RV ESA, mm^2^	10.7 ± 2.4	12.9 ± 2.9	0.033
FAC, %	45.3 ± 5.2	46.7 ± 7.5	0.583
Rest HR, bpm	76.8 ± 12.7	67.2 ± 13.8	0.031
Peak HR, bpm	164.9 ± 14.4	162.2 ± 9.4	0.420
MTHR, %	84.1 ± 7.1	83.3 ± 5	0.628
Rest SBP, mmHg	105.6 ± 12.9	112.8 ± 10.8	0.053
Rest DBP, mmHg	65.6 ± 7	70.3 ± 6.9	0.035
Peak SBP, mmHg	155.3 ± 19.4	178.3 ± 19.1	0.0002
Peak DBP, mmHg	70.6 ± 5.5	72.9 ± 8.8	0.355
Watt	179.7 ± 33.7	221.4 ± 47.9	0.003
W/Kg	2.53 ± 0.7	2.98 ± 0.5	0.011

Abbreviations: AA: ascending aorta; AR: aortic root; BMI: body mass index; BSA: body surface area; DBP: diastolic blood pressure; EDA: end-diastolic area; EDDi: end-diastolic diameter index; EDVi: end-diastolic volume indexed; EF: ejection fraction; ESA: end-systolic area; ESDi: end-systolic diameter index; ESVi: end-systolic volume index; FAC: fractional area change; HR: heart rate; IVS: interventricular septum; LA: left atrium; LAVi: left atrium volume index; LAX: long axis; LV: left ventricle; LVMi: left ventricular mass index; MTHR: maximal theorical heart rate; PASP: pulmonary artery systolic pressure; PW: posterior wall; RV: right ventricle; RVOT: right ventricle outflow tract; RWT: relative wall thickness; SBP: systolic blood pressure; TAPSE: tricuspid annular plane systolic excursion; W: watt.

**Table 2:** Types of left ventricle remodeling in power athletes according to sporting discipline.

		Weightlifting	Judo	*p*
N (%)	Global	17	38	
	Male	8	17	
	Female	9	21	
EH, (%)	Global	0 (0)	9 (23.7)	0.028
	Male	0 (0)	5 (29.4)	0.086
	Female	0 (0)	4 (19)	0.159
NG, (%)	Global	17 (100)	28 (73.7)	0.019
	Male	8 (100)	11 (64.7)	0.053
	Female	9 (100)	17 (80.9)	0.159
CH, (%)	Global	0 (0)	1 (2.6)	0.499
	Male	0 (0)	1 (5.9)	0.700
	Female	0 (0)	0 (0)	>0.99
CR, (%)	Global	0 (0)	0 (0)	>0.99
	Male	0 (0)	0 (0)	
	Female	0 (0)	0 (0)	

Abbreviations: CH: concentric hypertrophy; CR: concentric remodeling; EH: eccentric hypertrophy; NG: normal geometry.

**Table 3:** Comparison of cardiopulmonary exercise test (CPET) results between weightlifters and athletes practicing judo.

N = 31	Weightlifting	Judo	*p*
n (%)	7	24	-
Male, n (%)	4 (57.1)	12 (50)	0.789
Watt	194 ± 45.3	238.8 ± 41.3	0.009
VO^2^ mL/kg	35.1 ± 5.4	41.8 ± 5.2	0.012
RQ	1.09 ± 0.08	1.08 ± 0.06	0.804
VE/O2 LT1, mL/min	1828.7 ± 770.5	1955.7 ± 443	0.041
VE/O2 LT2, mL/min	2112 ± 399.7	2750.3 ± 511.9	0.036
VE/CO2 LT1	27.5 ± 3.9	25 ± 2.4	0.070
VE/CO2 LT2	29.3 ± 1.7	26.9 ± 2.5	0.129
O_2_ pulse	14.8 ± 2.7	19.2 ± 3.3	0.007

Abbreviations: LT1: first lactate threshold; LT2: second lactate threshold; O_2_ pulse (mL/beat): oxygen uptake per heartbeat, an indicator of stroke volume and oxygen extraction; RQ: respiratory quotient, the ratio of CO_2_ production to O_2_ consumption, indicating the predominant substrate for energy production; VE: minute ventilation; VO_2_ (mL/kg): oxygen uptake: maximal oxygen uptake normalized to body weight, a measure of aerobic capacity. VE/O2 and VE/CO2 at LT1 and LT2: ventilatory equivalents for O_2_ and CO_2_ at the first and second lactate thresholds, reflecting ventilatory efficiency.

## Data Availability

The raw data supporting the conclusions of this article will be made available by the authors on request.
